# Genomic Biomarkers of Meningioma: A Focused Review

**DOI:** 10.3390/ijms221910222

**Published:** 2021-09-23

**Authors:** Jacob A. Pawloski, Hassan A. Fadel, Yi-Wen Huang, Ian Y. Lee

**Affiliations:** 1 Department of Neurosurgery, Henry Ford Hospital, Detroit, MI 48202, USA; hfadel1@hfhs.org (H.A.F.); yhuang4@hfhs.org (Y.-W.H.); ilee1@hfhs.org (I.Y.L.); 2 Department of Neurological Surgery, Henry Ford Hospital, 2799 West Grand Blvd, Detroit, MI 48202, USA

**Keywords:** meningioma, genomics, biomarkers

## Abstract

Meningiomas represent a phenotypically and genetically diverse group of tumors which often behave in ways that are not simply explained by their pathologic grade. The genetic landscape of meningiomas has become a target of investigation as tumor genomics have been found to impact tumor location, recurrence risk, and malignant potential. Additionally, targeted therapies are being developed that in the future may provide patients with personalized chemotherapy based on the genetic aberrations within their tumor. This review focuses on the most common genetic mutations found in meningiomas of all grades, with an emphasis on the impact on tumor location and clinically relevant tumor characteristics. NF-2 and the non-NF-2 family of genetic mutations are summarized in the context of low-grade and high-grade tumors, followed by a comprehensive discussion regarding the genetic and embryologic basis for meningioma location and phenotypic heterogeneity. Finally, targeted therapies based on tumor genomics currently in use and under investigation are reviewed and future avenues for research are suggested. The field of meningioma genomics has broad implications on the way meningiomas will be treated in the future, and is gradually shifting the way clinicians approach this diverse group of tumors.

## 1. Introduction

Meningiomas are the most common tumors of the central nervous system, accounting for at least one-third of all intracranial tumors [1]. The World Health Organization (WHO) currently classifies meningiomas into one of three grades—WHO Grade 1 Benign, WHO Grade 2 Atypical, and WHO Grade 3 Anaplastic—with 15 further stratified subtypes based solely on the morphological features of the tumor cells with no consideration for molecular markers [2]. Factors differentiating between Grade 1–3 meningiomas are largely histomorphologic in nature and include: the degree of mitotic activity, the presence of brain invasion, and additional evidence of aggressive histologic activity [2,3]. Despite its widespread use, the WHO classification fails to accurately predict the clinical behavior, aggressiveness, and long-term recurrence of particular meningiomas [4,5,6,7]. The subjective diagnostic criteria of the WHO classification, relying heavily on vague histological analysis, has been shown to result in suboptimal interobserver variability and questionable prognostic value [6,8,9,10]. The shortcomings of the WHO classification can therefore hinder accurate diagnoses, misrepresent risk of recurrence, and leave clinicians with an insufficient tool to guide treatment decisions.

Technological advancements, paired with the rapidly expanding field of genomics, have led to an expanded and nuanced understanding of the oncogenesis and genomic profile of meningiomas. The recognition of the association between meningioma formation and neurofibromatosis 2 (NF-2) gene inactivation [11,12,13,14,15] over three decades ago has opened the door to the identification of countless genetic alterations of a growing number of non-NF-2 genes [16,17,18,19,20,21,22,23,24]. The identification of specific genetic alterations allows for further classification of meningiomas into genetic subgroups, thereby augmenting established histomorphologic classifications and providing the potential for more improved diagnosis, prognosis, and possible treatment. The following review discusses the genomic biomarkers of meningiomas, their clinical correlates, and corresponding targeted therapies.

## 2. The Genomics of Meningiomas 

**Figure 1:**
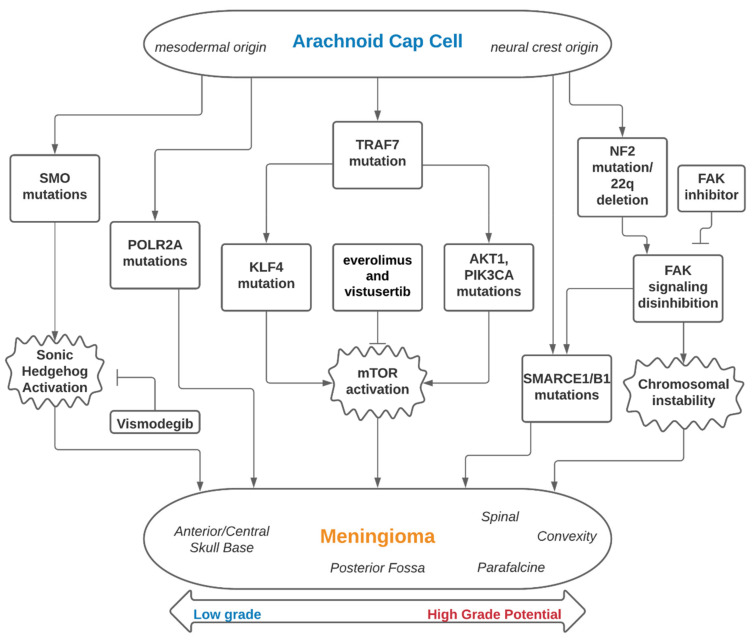
Embryology, genomics and therapeutic interventions of meningiomas.

The genomic landscape of meningiomas can be broadly classified into two subsets that focus on the involvement of the Neurofibromatosis type 2 (NF-2) gene—tumors associated with mutations of NF-2 and tumors with non-NF-2 mutations (Table 1). Building off of findings in the 1970s showing meningiomas to be associated with a deletion of Chromosome 22q, the NF-2 on 22q12 and its inactivation was later identified as a major driver of meningioma oncogenesis as well as the genetic alteration underlying the familial syndrome of neurofibromatosis type 2 [11,12,14,15,25,26]. Thanks to whole-genome sequencing technology powering the effort to further investigate the genetic underpinning of meningiomas, non-NF-2 genetic subtypes have since been identified, such as Tumor necrosis factor receptor-associated factor 7 (TRAF7), Kruppel-like factor 4 (KLF4), Smoothened, frizzled class G protein-coupled receptor (SMO), v-Akt murine thymoma viral oncogene homolog 1 (ATK1), and Phosphatidylinositol-4,5-Bisphosphate 3-Kinase Catalytic Subunit Alpha (PIK3CA) (Figure 1). We will review the genes and germline mutations associated with meningiomas and highlight their clinical importance and relevant research. 

### 2.1. NF-2 

NF-2 is located on chromosome 22q12.2 and codes for the Merlin protein which is a cytoskeletal protein and also has tumor suppressor activity. NF-2 is the most commonly associated gene associated with meningioma oncogenesis, with 50–60% of meningiomas having either an inactivating NF-2 mutation or monosomy of chromosome 22 with resultant loss of an NF-2 gene [28,31]. Germline mutations in NF-2 cause the inherited genetic disorder neurofibromatosis type 2, characterized by the development of schwannomas and meningiomas [28]. Given the strong and prevalent association between NF-2 mutations and meningiomas, some authors recommend germline NF-2 screening be done prior to meningioma resection in patients younger than 30 years old or with multiple central nervous systems tumors [26]. While NF-2-associated genetic alterations dominate the spectrum of meningioma biomarkers, the remaining biomarkers that have been identified can be grouped in an inclusive non-NF-2 family of meningioma mutations. These other mutations are less common, more heterogeneous, and often result in different tumor phenotypes than the NF-2-associated mutations. 

### 2.2. Non-NF-2 Genetic Subtypes

While often grouped together into the inclusive subgroup of non-NF-2 mutations, genomic and proteomic analyses have allowed for a more nuanced and rational classification of the heterogeneous non NF-2 genes involved in meningioma tumorigenesis. Of the non-NF-2 gene alterations associated with meningiomas, TRAF7 is the most common [24,26,28]. Found in over 50% of non-NF-2 tumors, TRAF7 mutations independently induce meningioma growth, but more commonly act in combination with one of several co-mutations including KLF4, and ATK1 [19,20]. ATK1 and PIK3CA are associated with the MTOR signaling pathway and act with TRAF7 in approximately 10–15% of all meningiomas and, interestingly, over 30% of all cancers [19,31,32,33]. AKT1 mutations occur at a conserved E17K location that results in activations of the mTOR and ERK1/2 signaling pathways which promote cell proliferation, and mTOR inhibitors have been suggested as adjunctive treatment for these cases [34]. Furthermore, SMO mutations as well as mutations of other downstream regulator proteins have been shown to induce meningioma formation via disinhibition of the Sonic hedgehog signaling pathway [35]. SMO and AKT1 in particular are primarily found in meningothelial or transitional WHO grade I meningiomas, are rare in WHO grade II lesions, and absent in WHO grade III meningiomas [24,28,36,37]. 

### 2.3. Germline Mutations

Other rarer germline mutations include SWI/SNF Related, Matrix Associated, Actin Dependent Regulator Of Chromatin, Subfamily B, Member 1 (SMARCB1), SMARCE1, BAP1, and SUFU genes. SMARCB1 and SMARCE1 are frequently reported in familial syndromes with multiple meningiomas [30]. Studies show that these germline mutations are more likely to present in younger patients with meningiomas and therefore, some recommend that younger patients and those with a family history of meningioma should obtain a germline mutation screen before resection [26]. Additionally, germline BAP1 mutations are usually associated with aggressive rhabdoid meningioma and are also linked with BAP1 tumor predisposition syndrome. Therefore, it is recommended that patients with germline BAP1 mutations and history of BAP1 tumor predisposition syndrome should obtain frequent surveillance images for meningioma [26].

### 2.4. High-Grade Meningiomas

The most common genetic driver mutations of low-grade meningiomas discussed above are much less frequently identified in high-grade tumors, with the exception of NF-2 [38]. Bi et al. demonstrated that in a sample of high-grade meningiomas the rate of NF-2 mutation was significantly elevated to 80% compared to low-grade tumors in which approximately 40% carry NF-2 mutations. Because the majority of non-NF-2 meningioma driver mutations such as SMO, AKT1, SMARCB1, and TRAF7 normally occur mutually exclusive of NF-2, the expected rate of these mutations would be lower in high-grade tumor cohorts. However, even among the 20% of high-grade tumors that did not harbor NF-2 mutations, the rate of occurrence of the non-NF-2 meningioma driver mutations was less than 5%, compared to 35% in low grade non-NF2 tumors. This significantly less frequent occurrence suggests a different genetic basis for many high-grade meningiomas. Sporadic somatic mutations are much more frequent in high-grade meningiomas than low-grade tumors, with an average of >20 somatic mutations per sample [38]. The results suggest that the inciting event for many high-grade meningiomas is a copy number loss of chromosome 22, which in addition to containing the tumor suppressor gene NF-2 may also contain other tumor suppressor genes such as SMARCB1, CHEK2, and CLH22 [39]. The loss of chromosome 22 creates a state of genetic instability in which a somatic mutations occur readily and lead to a genetic hetergenous aggressive tumor phenotype. Further research will be needed to describe why many NF-2 mutated meningiomas with chromosome 22 deletion do not experience a transformation to high-grade histopathology.

Chromosomal abnormalities beyond chromosome 22 deletions are expectedly much more common among high-grade tumors, and the variability in meningioma chromosomal aberations can in some ways aid in understanding the pathogenesis of malignant phenotypes in meningioma. Chromosomal studies have shown that the majority of NF-2 mutated meningiomas are accompanied by marked chromosomal instability that produced copy loss or duplication of a multitude of other chromosomes [40]. However, there are also a subgroup of non-NF2 mutated tumors that also display a high level of chromosomal instability. Van Tilborg et al. conducted chromosomal studies on 61 meningiomas and identified 4 distinct cohorts determined by the presents or absence of NF-2 mutations and the amount of chromosomal instability, determined by the standard deviation of chromosomes between different metaphase samples from the same tumor specimen. While NF-2 mutations were associated with significantly more clonal chromosomal variability compared to non NF-2 mutated tumors, there was a subgroup of non-NF2 mutated tumors with significant heterogeneity in copy number between different metaphases within the same sample [40]. This suggests that there are influences beyond the activity of the merlin gene that result in a higher degree of chromosomal instability. Variability in the genetic basis behind chromosomal abnormalities also could explain why many tumors do not make high-grade transformations despite having significant chromosomal structure abnormalities. 

One clinical challenge created by the genetic landscape of high-grade tumors is that the majority of targeted therapies for meningiomas focus on interrupting oncogenic pathways associated with non-NF-2 mutations (as will be discussed later). Since high-grade mengingiomas often harbor a diverse and variable group of somatic mutations it is increasingly difficult to identify therapeutic targets for these tumors. As will be discussed later, there are clinical trials ongoing utilizing FAK inhibitors in NF-2 mutated tumors due to the importance of FAK signaling in the tumor suppressor activity of NF-2. mTOR signaling has also been identified as a target of Merlin inhibition, and therefore mTOR inhibitors are being studied shown to be effective at inhibiting growth of meningioma cells in animal models [41]. More recently, human clinical trials utilizing mTOR inhibitors have begun enrolling patients including a phase II study examining everolimus and octreotide in patients with recurrent mostly grade II or III meningiomas [42]. This study demonstrated an average decrease in tumor growth rate from 16.6% increase in size per three months prior to treatment to 0.48% per three months at six months post-treatment initiation, with 78% of tumors experiencing at least 50% reduction in growth rate. Four patients in the study had NF-2 germline mutations.

Programmed death-ligand 1 (PD-L1) is a cell surface receptor used by a variety of cancer cells to evade immune-mediated apoptosis of the malignant cells, and PD-L1 inhibitors such as Pembrolizumab have been approved for treatment of multiple cancer types including melanoma, and lung and head/neck carcinomas. PD-L1 has also been shown to be expressed in meningiomas and has been associated with poorer prognosis and higher histologic grade with up to 88% of high-grade meningiomas expressing PD-L1 [43]. There are several phase II clinical trials ongoing evaluating PD-L1 and other immune checkpoint inhibitors in high-grade and recurrent meningiomas, and this will hopefully shed light on the efficacy of these immunotherapies in meningioma. Additional investigational therapies for low- and high-grade meningioma is discussed in depth below.

### 2.5. Meningioma Epigenetics and Serum Biomarkers

As is also true in other tumor types, meningioma development has both genetic and epigenetic implications. One of the best characterized epigenetic changes associated with tumorogenesis is the alteration of DNA methylation patterns. In tumor cells this results in a globally hypomethylated genome with select areas of hypermethylation around specific DNA promoter regions resulting in altered gene expression [44,45]. Abnormal methylation of conserved CpG islands in cancer cell DNA has been identified as a key epigenetic signature that is often unique to different cancer types [46]. Studying DNA methylation alterations in meningiomas has resulted in new classification schemes that account for variable methylation profiles [47,48]. Nassiri et al. demonstrated that methylation profiles of specific genes in meningiomas has been shown to correlate with shorter time to recurrence [49]. Altered methylation patterns have also been studied in plasma cell-free DNA, and CNS tumors including meningiomas have also been accurately categorized based on DNA methylation profiles acquired from serum samples [48,50]. This area of active investigation has great clinical potential, offering the ability for minimally invasive detection and identification of CNS tumors including meningioma via a simple blood draw. 

## 3. Genetic Biomarkers and Tumor Location

**Figure 2:**
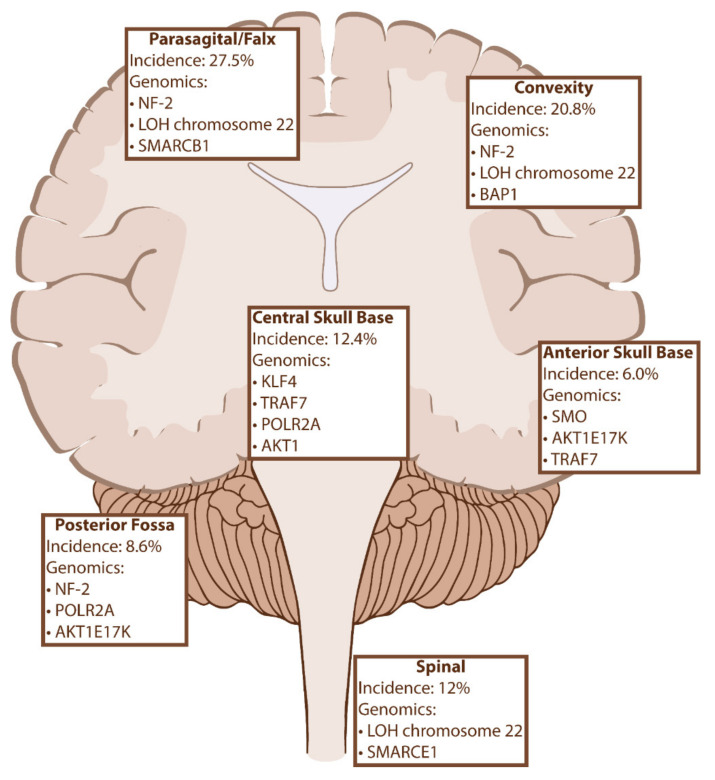
Meningioma location and related genomic markers with incidences [51,52]. Abbreviations: LOH, Loss of Heterozygosity; NF-2, neurofibromatosis 2; TRAF7, Tumor necrosis factor receptor-associated factor 7; KLF4, Kruppel-like factor 4; AKT1, v-Akt murine thymoma viral oncogene homolog 1; SMO, Smoothened, frizzled class G protein-coupled receptor; POLR2A, enzyme RNA polymerase II, subunit A; SMARCB1, SWI/SNF-Related, matrix-associated, actin-dependent regulator of chromatin subfamily B member 1; SMARCE1, SWI/SNF Related, matrix-associated, actin-dependent regulator of chromatin subfamily E member 1.

The previously discussed genetic biomarkers of meningiomas not only influence tumor oncogenesis and behavior but are increasingly being recognized for their influence on tumor location (Figure 2). While there is significant overlap between the various mutations, there is a clear correlation between certain genes and their favored locations in the cranial vault. For example, NF-2 mutations can be found anywhere throughout the CNS, they have a propensity to be implicated in tumors of the convexity and posterior skull base and are exceedingly less common than mutations in tumors of the anterior and medial skull base. As we will discuss, these trends also impact histopathology, prognosis, and recurrence rates, suggesting that there is a complex biomolecular landscape of meningiomas that is not captured in the current WHO classification.

### 3.1. Anterior Skull Base

Meningiomas of the midline anterior skull base have been shown to harbor a variety of mutations but two are most common: missense mutations in SMO and the single activating mutation AKT1E17K [19,24,28]. SMO mutations result in disinhibition of Sonic hedgehog signaling and are most frequently found in tumors of the anterior skull base (20–30% of olfactory groove meningiomas), while AKT1 mutations can result in anterior skull base, clival or convexity locations [19,24,28,31,35]. Although tumors associated with either mutation are primarily WHO grade 1 meningothelial subtype, SMO mutations have been associated with significantly higher recurrence rates at 10 years compared to AKT1 and other oncodriver mutations after controlling for Simpson grade and WHO grade [36]. While AKT1E17K is associated with a TRAF7 mutation approximately 60% of the time, SMO mutations are independently tumorigenic [19]. TRAF7 mutations can also be found in meningiomas of the anterior skull base and middle cranial fossa in absence of any other oncogenes, suggesting an independent role or the presence of unidentified genetic drivers [53].

### 3.2. Central Skull Base

KLF4 is a member of a family of DNA-binding transcription factors associated with cell cycle progression and inflammatory processes [27,54]. KLF4K409Q is the only mutation identified in the KLF4 gene associated with meningiomas, and like AKT1, it is commonly associated with TRAF7 co-mutation in meningiomas of the central skull base and sphenoid wings. In addition to tumor location, KLF4/TRAF7 mutations are independently correlated with secretory histopathology, with nearly all secretory meningiomas harboring this combination of mutations [29,53]. POLR2A mutations also predispose to parasellar skull base meningiomas and independently account for approximately 4–6% intracranial lesions [20,31]. Like SMO, POLR2A-associated tumors are nearly exclusively characterized as meningothelial grade I on histopathology. AKT1 mutations are also associated with antero-lateral or central skull base locations [31]. 

### 3.3. Parafalcine

The majority of convexity and parasagittal meningiomas are associated with NF-2 mutants or chromosome 22 deletions [31]. In addition to NF-2, SMARCB1 is now recognized as a driver in meningioma development with a strong predilection for the anterior falx cerebri region [55,56]. SMARCB1 resides on chromosome 22q11 and codes for a chromatin remodeling protein subunit [55,56]. Germline mutations in SMARCB1 have been associated with familial schwannomatosis, falcine meningiomas and rhabdoid tumor formation [55,56]. As many as 70% of NF-2-associated meningiomas of the anterior falx have SMARCB1 co-mutation, compared to 16% co-mutation rate in tumors of other locations [20]. The tumors are thought to develop via loss of heterozygosity of NF-2 and SMARCB1 whereby the cells lose one copy of chromosome 22 and develop loss of function mutations in the remaining copy of each gene [56]. SMARCB1 has also been associated with increased Ki-67 index, which is consistent with their occasional propensity for atypical pathology [53,57].

### 3.4. Posterior Fossa

Surgical resection for posterior fossa meningioma is challenging due to the adjacency of critical neurovascular structures and complex anatomy of the temporal bone [58]. Due to a more challenging surgical approach and difficulty in removing affected dura/bone from the posterior skull base, incomplete resection, and tumor recurrence are not uncommon [34]. A recent large genetic analysis found high rates of NF-2 and POLR2A alterations in posterior fossa region meningiomas [53]. In addition to the anterior skull base, AKT1E17K mutations were also found in posterior fossa and specifically as many as 50% of foramen magnum meningiomas in one series [59]. Identifying genetic biomarkers for these difficult to treat tumors is especially important as this may lead to potential therapeutic targets for tumors that recur or are incompletely resected.

### 3.5. Spinal

Spinal meningiomas are less common than other anatomical locations, thus there are fewer genomic studies available that examine genetic biomarkers in this tumor subset. Monosomy 22 is the most common genetic abnormality in spinal meningiomas, with over 70% containing chromosome 22 alterations [60,61]. In addition to NF-2 associated alterations, SMARCE1 (Chr. 17q21) encodes another chromatin-remodeling complex subunit and has been associated with tumor suppressor functions and implicated in the development of spinal and cranial clear cell meningiomas [22,62]. Several different loss of function mutations in SMARCE1 have been described, and germline mutations in SMARCE1 were associated with a familial multiple spinal meningioma syndrome [22]. With the exception of SMARCE1 mutant tumors, psammomatous, transitional and meningothelial are most common histopathology of spine meningioma. Transitional and psammomatous are more common in spinal than cranial tumors, but the genetic basis for this finding is unclear [61].

## 4. Embryologic Basis for Meningioma Heterogeneity

When discussing the anatomic localization of various meningioma biomarkers, the majority of studies are in agreement on which genetic mutations predispose to tumors in certain locations. Lacking in our current understanding of meningioma tumorigenesis is the basis for which specific mutations have a propensity for occurring repeatedly in stereotypical locations. It has recently been hypothesized that the primary driver behind this phenomenon is the embryologic origins of the meninges from which these tumors develop [31]. Anatomical and embryologic studies in humans and animals indicate that the dura that comprises the posterior fossa primarily originates from dorsal mesoderm, where as the falx and frontal convexity dura stems from the neural crest, and medial skull base dura from paraxial mesoderm [63]. By categorizing meningiomas based on the presumed embryologic origin, Okano et al. demonstrated that NF-2 mutant tumors likely originate from neural crest-derived arachnoid cells, while non-NF-2 tumors stem from dorsal and paraxial mesoderm. This hypothesis of embryologic determinism offers an enticing explanation as well for the differential trends in histopathology among the various locations. 

A different embryologic origin could explain why the non-NF-2 associated meningiomas are almost always benign, heavily favor meningothelial histology, and do not experience the multiple chromosomal aberrations that are common with the majority of NF-2 tumors [19,24,28]. The exception is tumors with PIK3CA mutations which do commonly display chromosomal aberrations, but when combined with TRAF7 mutations this results in less chromosomal instability [16]. Conversely, atypical and anaplastic meningiomas are disproportionately associated with NF-2 mutations and/or 22q chromosome deletions, which may relate to genetic factors associated with a mesodermal origin. 

Taken together, the embryologic origin hypothesis allows for conceptualizing the multitude of meningioma histologies and genetic biomarkers into two general subtypes: an NF-2/22q deletion family, comprising tumors originating from the neural crest with more potential to develop atypical features, and a non-NF-2 family originating from paraxial mesoderm that are generally benign and of the meningothelial variety. As discussed previously, the non-NF-2 family is actually composed of a heterogenous mix of genetic alterations that can be localized to mTOR, PI3K, and Sonic Hedgehog signaling pathways, but these most frequently produce benign tumors. High-grade meningiomas are nearly exclusively associated with NF-2 mutations or q22 deletions, with only 5% of high-grade meningiomas harboring mutations in mTOR, Sonic hedgehog or other known tumorigenic pathways [38]. This underscores the genetic instability produced by NF-2 alterations, but may also be a reflection of inherent genetic instability in the arachnoid cells derived from the neural crest, with potential for further chromosomal aberrations and malignant transformation. Future studies are needed to fully investigate this theory. 

Genetic biomarker analysis has demonstrated that tumor recurrence is not strictly correlated to tumor histological grade but rather that individual mutations can independently increase the risk of tumor recurrence. For example, POLR2A mutations have been shown to be highly correlated with increased recurrence rate, while at the same time being exclusively of the meningothelial variety. Although POLR2A tumors were more commonly subtotally resected due to their location in the central skull base, the recurrence rate of 29% was shown to be significant even when controlling for the Simpson grade of resection [31].

## 5. Targeted Therapies for Meningioma

Several different therapeutic targets have been identified to target the previously discussed genetic biomarkers in meningiomas, opening the door for targeted and personalized treatment options for patients. There are currently a number of early phase clinical trials focused on targeted therapies for meningiomas (Table 1) [64]. Vismodegib is an inhibitor of the sonic hedgehog signaling pathway that is FDA approved for treatment of basal-cell carcinoma, but is under investigation in several other tumor types including in SMO-related progressive meningiomas (ClinicalTrials.gov Identifier: NCT02523014, accessed 14 April 2021). 

In one case report, the AKT1 inhibitor AZD5363 was associated with modest tumor regression and prolonged local control when used as monotherapy in a case of recurrent meningioma with documented AKT1 mutation [56]. The same case was also noted to have metastatic meningioma nodules in bilateral lungs and these lesions were also found to be stable after AKT1 inhibitor use. mTOR pathways, which are activated by meningiomas with ATK1 and PIK3CA mutations, are also being targeted by mTOR inhibitors everolimus and vistusertib (NCT03071874, NCT01880749). The advantage of these therapies is that mTOR inhibitors are already in use for other solid tumors and are therefore better understood in terms of their dosing, pharmacodynamics, and safety.

Focal adhesion kinase (FAK) is a protein tyrosine kinase that integrates intracellular signaling and influences cell proliferation [65]. The tumor suppressor activity of NF-2 is mediated in part by downregulating interactions with FAK signaling, and tumor cells with NF-2 inactivation or q22 deletion have been shown to respond to FAK inhibition [66]. FAK inhibitor GSK2256098 is currently under investigation for its role in treating NF-2 mutation-associated meningiomas (NCT02523014). 

In addition to a number of therapies specific to genetic mutations, there are several avenues for targeted therapies for meningiomas that do not rely on a specific mutation. Hormonal therapies and immunotherapies have potential for efficacy in a broader range of meningiomas dependent on the level of expression of various cell surface receptors in a given tumor. Estrogen and progresterone receptors are upregulated in many meningioma cells accounting for the higher frequency among females and the increase in growth during pregnancy. The anti-progesterone medication mifepristone was investigated using a randomized, controlled trial but no significant difference in overall survival or progression free survival was identified [67]. Octreotide, a somatostatin analog, has been studied in both recurrent grade 1 as well as atypical and anaplastic meningiomas and demonstrated mixed results although low-grade tumors seemed to respond well to treatment with 100% progression free survival at 48 months [68].

Vascular endothelial growth factor (VEGF) is produced by many cancers as a means of stimulating intratumoral angiogenesis, including in meningioma. VEGF has been well-studied in a variety of cancers and VEGF inhibitors such as bevacizumab are frequently utilized in other intracranial malingnancies. VEGF studies in meningioma have demonstrated promising results with one study finding an overall progression free survival of 18 months among mixed cohort of 14 tumors of all grades [69]. Other growth factors such as platelet-derived growth factor (PDGF) and epidermal growth factor (EGF) also have increased expression in meningioma, and stand as possible future therapeutic targets. PDGF is inhibited by imatinib and has demonstrated some ability to slow meningioma progression, although with mixed results. In a small cohort of nine patients with recurrent meningioma treated with imatinib there was an overall median progression free survival of 16 months [70].

Immunotherapeutics such as PD-1 and PD-L1 inhibitors have been studied and have shown early signs of activity against certain meningioma genotypes. PD-1 inhibitors pembrolizumab and nivolumab are currently being investigated for their role in high-grade meningioma because it has been demonstrated that anaplastic meningiomas strongly express PD-1 and PD-L1 [71]. Tumor treating fields is a treatment modality currently employed in glioblastoma which utilizes electrical fields delivered via electrodes on the patient’s head to disrupt cell division in cancer cells. NCT02847559 is an open-label study evaluating the application of tumor treating fields via Optune device in malignant meningiomas.

## 6. Conclusions

Genomic research over the past decade has shed new light on the heterogeneous genetic landscape of meningiomas, such that tumor location, histopathology, prognosis and response to treatment can now be explained and informed by an understanding of the combination of oncogenes that drive each tumor. Precise identification of aggressive tumors with a high risk of recurrence is essential to clinical decision-making and to have informed discussions with patients about their prognosis. Although, at present, only pathologic features define WHO grading, it is likely that upcoming WHO brain tumor classification updates will incorporate molecular assessment as part of the integrated diagnosis of meningioma grade. The next decade will look to determine which genetic mutations impart an increased risk of tumor recurrence, and expand targeted therapies for recurrent or high-grade meningiomas based on tumor genomics. A deeper understanding of genetic factors at play in atypical and anaplastic tumors will be critical to treating the most aggressive tumor pathologies and improving survival rates of meningioma patients.

## Figures and Tables

**Table 1:** Gene alterations of meningiomas with corresponding WHO grade, location, and potential therapies. Abbreviations: NF-2, neurofibromatosis 2; TRAF7, Tumor necrosis factor receptor-associated factor 7; KLF4, Kruppel-like factor 4; ATK1, v-Akt murine thymoma viral oncogene homolog 1; PIK3CA, Phosphatidyl-inositol-4,5-Bisphosphate 3-Kinase Catalytic Subunit Alpha; SMO, Smoothened, frizzled class G protein-coupled receptor; POLR2A, enzyme RNA polymerase II, subunit A; SMARCB1, SWI/SNF-Related, matrix-associated, actin-dependent regulator of chromatin subfamily B member 1.

Gene	WHO Grade	Tumor Location	Targeted Therapy
NF-2	I–III	Parafalcine [9,21] Posterior fossa [27] Spine [28]	Focal Adhesion Kinase (FAK) inhibitor GSK2256098 (NCT02523014)
TRAF7	I–III	Anterior Skull Base [27] Middle Cranial Fossa [27,29] Sphenoid Wing [27,29]	
KLF4	I	Central Skull Base [27,29]	
AKT1	I	Anterior Skull Base [8,18,21] Central Skull Base [21] Posterior Fossa [30]	mTOR inhibitors everolimus NCT03071874 vistusertib NCT01880749
PIK3CA	I–III	Anterior & Middle Skull Base [1]	mTOR inhibitors everolimus NCT03071874 vistusertib NCT01880749
SMO	I	Anterior Skull Base [8,29]	Vismodegib NCT02523014
POLR2A	I	Parasellar [9,21]	
SMARCB1	I–III	Parafalcine [9] Spine [28]	
